# Modeling the Potential Distribution of the Invasive Pest *Trogoderma granarium* (Coleoptera: Dermestidae) Under CMIP6 Future Climate Projections

**DOI:** 10.1002/ece3.72159

**Published:** 2025-09-16

**Authors:** Chao Zhao, Duangsamorn Suthisut, Chunqi Bai, Lei Yan, Dianxuan Wang, Liang Chen, Jianhua Lü, Liang Li, Peng Li

**Affiliations:** ^1^ National Grain Industry (Storage Insect Pest Control) Technology Innovation Center Henan University of Technology Zhengzhou China; ^2^ Grain Storage and Logistics National Engineering Research Center Henan University of Technology Zhengzhou China; ^3^ Industry & Technology Innovation Center of Green‐Intelligence Control of Stored Products Pests Henan University of Technology Zhengzhou China; ^4^ Postharvest Technology Research and Development Group Postharvest and Processing Research and Development Division, Department of Agriculture Bangkok Thailand; ^5^ School of Food and Strategic Reserves Henan University of Technology Zhengzhou China; ^6^ Shaanxi Xirui (Group) Co., Ltd Xi'an China; ^7^ Institute for Complexity Science Henan University of Technology Zhengzhou China

**Keywords:** CMIP6, ecological niche models, MaxEnt, stored product pest, suitable habitat

## Abstract

The Khapra beetle ( *Trogoderma granarium* Everts) is a well‐known storage pest, and it is listed as one of the 100 most invasive species in the world. This study predicted the potential geographic distribution of the pest based on the MaxEnt model and assessed the impact of environmental factors on distribution patterns. Results indicate that under current climate conditions, central and southern China, the southern United States, North Africa, southern Australia, and Argentina are highly suitable regions for *T. granarium* . The minimum temperature of the coldest month and elevation are the primary influencing factors, contributing over 95% to the model. Projections for the future under SSP126 and SSP585 scenarios suggest no significant expansion in total suitable habitat. However, binary mapping and centroid analysis indicate a northward shift in the species' suitable range, with new suitable areas emerging primarily in the northern United States, westernmost Russia, southern Kazakhstan, and northern China. These findings offer crucial data that can support the development of pest monitoring systems, early warning protocols, and quarantine strategies in countries at high risk.

## Graphical abstract

The significant invasive pest, *Trogoderma granarium* , is currently recorded to have a limited distribution range. However, our study reveals that under current and future climatic conditions, this pest possesses an extensive potential distribution worldwide, with China's central and southern regions, southern USA, North Africa, southern Australia, and Argentina identified as high‐risk countries.

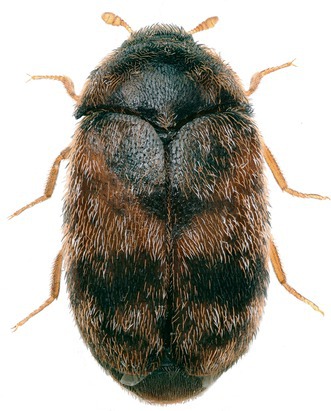

## 1 Introduction

**FIGURE 1:**
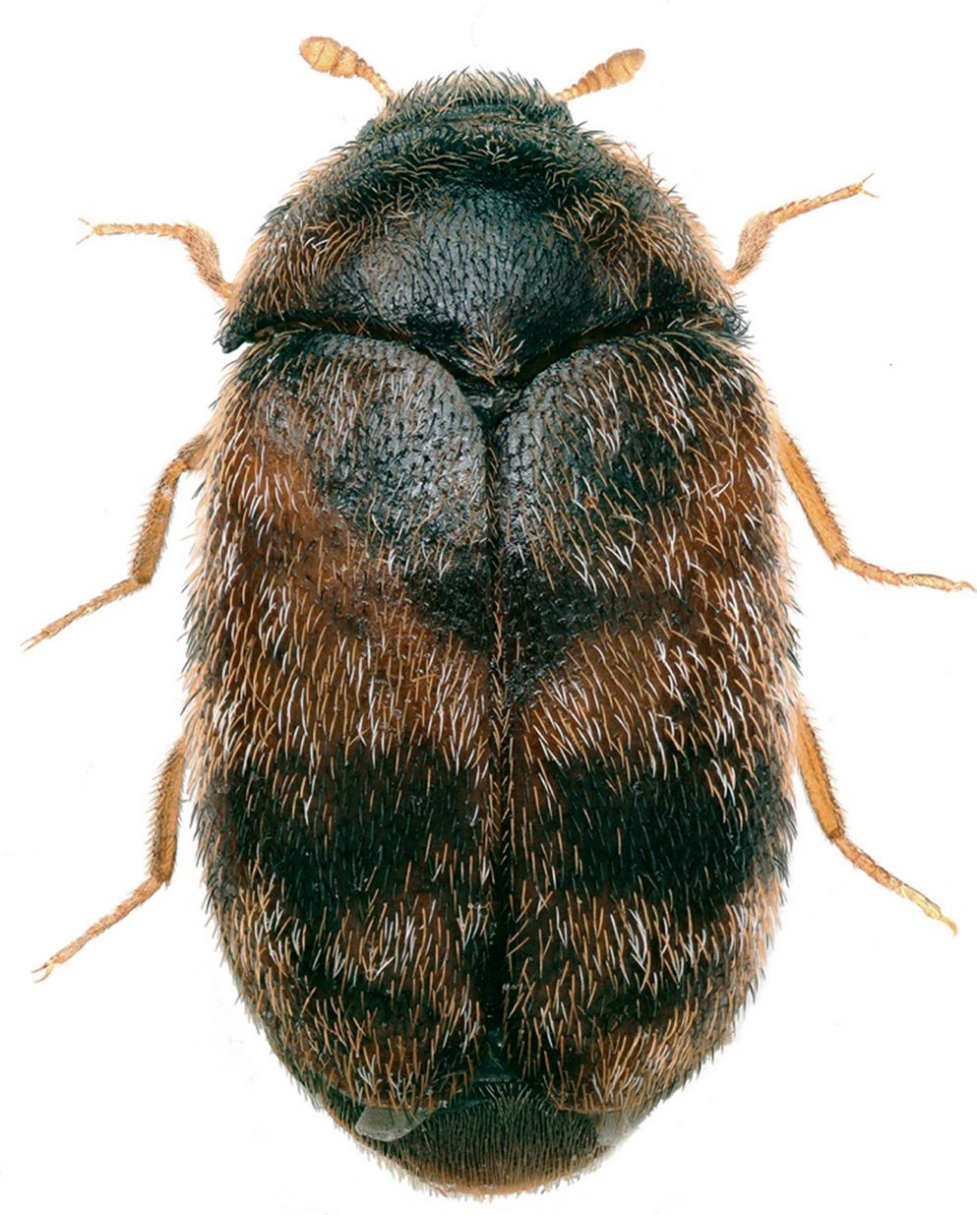
*Trogoderma granarium* (photograph by Xuguang Bai).

*Trogoderma granarium* Everts (Figure 1), commonly referred to as the Khapra beetle, is listed among the world's top 100 invasive species (Lowe et al. 2000) and is designated as an A2 quarantine pest by the European and Mediterranean Plant Protection Organization (EPPO 2024). Numerous studies have shown that *T. granarium* is a highly polyphagous pest of stored products, capable of infesting over 100 types of commodities (Pasek 1998; Hagstrum and Subramanyam 2009), including various grain seeds, legumes, nuts, and dried plant or animal products. Extensive feeding and fecal contamination by *T. granarium* cause significant weight and quality losses in stored grains, posing a severe threat to global food security (Ahmedani, Shaheen, et al. 2007). Although its global distribution is relatively limited, the species possesses remarkable adaptability and survival abilities. Once introduced into a new area, the pest can rapidly reproduce and spread under favorable conditions, with larvae capable of surviving prolonged periods through diapause, even under unfavorable conditions (Burges 1959, 1962, 1963). To protect national production and prevent the further spread of *T. granarium* , the World Trade Organization has banned the import of wheat, corn, and other grains and their processed products infested by this pest.

The Khapra beetle is believed to have originated in the Indian subcontinent and was first reported as a pest in 1894 (Barnes and Gbove 1916). To date, this pest has established populations in 34 countries across Asia, Africa, and Europe (Athanassiou et al. 2019). Additionally, records indicate that the species has invaded 32 other countries, but these invasions were eventually eradicated or died out naturally (Athanassiou et al. 2019). The pest prefers hot and dry climates, with its habitats primarily located between 35° N and 35° S (Day and White 2016). Notably, many countries that have successfully eradicated the pest (mostly in Europe) are outside this climatic zone. It remains unclear whether the successful eradication in these countries is due to unsuitable geographic and climatic conditions or effective eradication measures. Moreover, as global climate warming continues, there is uncertainty about how the global distribution of *T. granarium* will change. Currently, there is a significant lack of research on the scientific quantitative analysis of the pest's global distribution, despite its critical importance for developing quarantine measures and strategies worldwide.

Ecological niche models (ENMs) estimate suitable habitats by analyzing the environmental data occupied by the target species. While several models have been developed to predict species distribution under climate change scenarios, including mechanistic models such as CLIMEX, these approaches often rely on extensive species‐specific ecological and physiological data that are rarely available for storage pests. In contrast, MaxEnt is a machine‐learning‐based niche modeling algorithm that performs robustly with presence‐only data and limited environmental information. The Maximum Entropy Species Distribution Model (MaxEnt) is particularly effective for estimating habitat suitability (Phillips et al. 2006, 2017). This model predicts potential geographic distribution patterns using presence‐only data, accommodates both continuous and categorical variables, and applies predictions swiftly across different geographic regions. MaxEnt can accurately assess species and sample sizes even in cases of small sample quantities (Elith et al. 2006; Peterson et al. 2007; Hernandez et al. 2008). Therefore, MaxEnt is widely used in studies predicting potential distributions and conducting risk assessments of various insects and other organisms (Zhang et al. 2022; Lin et al. 2024).

Although several studies have assessed the global suitability of multiple invasive pests using MaxEnt (Cao and Feng 2024), such works often apply a uniform modeling framework and lack ecological refinement at the species level. This study focuses on *T. granarium* , a high‐risk quarantine pest, and aims to generate high‐resolution distribution models under current and future climate conditions across multiple SSP scenarios. In addition to rigorous parameter tuning and variable evaluation, we conducted region‐specific sensitivity analyses of potential distributions and proposed targeted management strategies for high‐risk areas. Beyond its academic significance, this research also aligns with several United Nations Sustainable Development Goals (SDGs), particularly SDG 2: Zero Hunger. By providing scientific evidence for early warning and pest control, the study contributes to the protection of global food security in the face of climate change.

## 2 Methods

### 2.1 Occurrence Data

In the initial survey, a total of 502 occurrence records of *T. granarium* were obtained from various sources, including literature, GBIF, CABI, EPPO, and survey reports. Records specifying only the country without precise locality information were excluded, while those indicating specific cities, counties, or towns were retained. Records with clear locality information, such as town names, were carefully verified and converted to coordinates using the Baidu Map API. To ensure data quality beyond geolocation cleaning, we removed duplicate records, verified suspicious entries against authoritative sources, and excluded points with environmental conditions inconsistent with the biology of *T. granarium* . The occurrence records were cleaned using the “Coordinate Cleaner” function from the R package, which helps remove inaccurate or erroneous coordinates. All occurrence coordinates were standardized to decimal degrees with a precision of three decimal places, corresponding to a spatial resolution of approximately 111 m.

To minimize spatial autocorrelation and sampling bias, the occurrence points were filtered using the approach outlined by Brown (2014). We applied a 7 km thinning threshold to all occurrence points. This threshold was selected based on two main considerations: (1) species‐specific studies on *T. granarium* dispersal within and around storage systems (Banks 1977; Athanassiou et al. 2019) indicate that its natural dispersal is very limited, typically confined to short distances between storage facilities; and (2) a 7 km distance is widely adopted in species distribution modeling literature to balance data independence and sample retention (Muscarella et al. 2014). This spatial thinning helps mitigate the clustering bias caused by dense sampling around trade hubs or storage sites, which often reflects sampling effort rather than the species' true ecological dispersal capacity. After filtering, only one point was retained per cluster, resulting in a final occurrence dataset comprising 89 unique point locations (see Table S1).

### 2.2 Environmental Data

Bioclimatic data of current and future were sourced from WorldClim (https://www.worldclim.org). Among them, current bioclimatic data reflect averages from 1970 to 2000, while future bioclimatic data represent periods of the 2050s (2041–2060), 2070s (2061–2080), and 2090s (2081–2100). Future climate data were selected from the Beijing Climate Center Climate System Model 2 Medium Resolution (BCC‐CSM2‐MR) in the Coupled Model Intercomparison Project Phase 6 (CMIP6). The future climate data from CMIP6 mainly include four shared socioeconomic pathways (SSPs), which represent different forcing scenarios: low forcing scenario (SSP126), medium forcing scenario (SSP245), medium to high forcing scenario (SSP370), and high forcing scenario (SSP585). In this study, SSP126 and SSP585 were selected to represent the lowest and highest expected levels, respectively. Although WorldClim data are available at 30 arc‐seconds (~1 km), we used 5 arc‐minutes (~10 km) resolution to maintain consistency with other studies on long‐distance pest invasions and to reduce computational complexity across large spatial and temporal extents. This coarser resolution also minimizes spatial uncertainty introduced by varying geolocation precision in historical occurrence records.

Correlation and multicollinearity among climate variables can negatively impact the predictive performance of niche models (Heikkinen et al. 2006). To reduce overfitting and improve model accuracy, Pearson correlation coefficients were used to assess the relationships between pairs of variables (see Table S2). Multicollinearity was identified using ENM Tools (version 1.0.40). The initial model assessed the contribution of various factors, followed by a correlation analysis. For pairs with correlation > 0.8, the variable with the higher contribution and greater biological relevance was selected. Table S2 presents the full correlation matrix, with all variable pairs above this threshold highlighted in red, and indicates the “Status” (“Retained” or “Removed”) of each predictor after filtering. Model performance improved slightly after variable selection (AUC increased from 0.873 to 0.889; TSS from 0.701 to 0.718). To ensure ecological relevance in addition to statistical independence, the final set of selected variables was further interpreted based on the known biology and environmental preferences of *T. granarium* . Specifically, Bio6 (minimum temperature of the coldest month) is highly relevant to larval survival, as the species can enter diapause to withstand low temperatures, but survival drops significantly below 0°C. Bio14 (precipitation of the driest month) reflects the extreme aridity this pest can tolerate, with known infestations occurring in grain products with moisture contents as low as 2%–3%. Bio15 (precipitation seasonality) indicates variation in humidity, which may influence development rates and infestation timing in both natural and storage environments. Elevation also plays an indirect role by affecting local temperature and humidity, both of which constrain the beetle's range. The inclusion of these variables thus aligns with both statistical rigor and biological plausibility. The final variables chosen were Bio6, Bio7, Bio8, Bio9, Bio14, Bio15, Bio18, Bio19 (Table 1), and elevation.

**TABLE 1:** Climatic variables used in the MaxEnt model.

Variable	Description	Unit
Bio6	Minimum temperature of the coldest month	°C
Bio7	Temperature annual range	°C
Bio8	Mean temperature of the wettest quarter	°C
Bio9	Mean temperature of driest quarter	°C
Bio14	Precipitation of the driest month	mm
Bio15	Precipitation seasonality (coefficient of variation)	—
Bio18	Precipitation of the warmest quarter	mm
Bio19	Precipitation of the coldest quarter	mm

### 2.3 Model Optimization and Settings

The modeling was performed using MaxEnt software (version 3.4.4k) with specific parameters: logistic output format, 25% of data used for random testing, up to 5000 iterations, a convergence threshold of 0.0001, 10‐fold cross‐validation, and a limit of 10,000 background points. Additionally, the settings included generating response curves and conducting a jackknife test to evaluate variable importance.

Model performance is affected by settings such as the regularization multiplier (RM) and feature combination (FC) (Phillips et al. 2017). Selecting appropriate parameters is crucial to avoid models being too simple or excessively complex (Morales et al. 2017). The R package “ENMeval” was utilized to select the optimal RM and FC settings, which automatically compute models based on specified parameter ranges (Muscarella et al. 2014).

A total of 116 candidate models were generated by pairing eight RM values (from 1 to 4, incremented by 1) with 31 possible combinations of feature classes, including all permutations of L, Q, P, T, and H. By comparing the AICc values under different combinations of regularization multiplier (RM) and feature classes (FC) (see Table S3), the model with RM = 3 and FC = QH was selected as the optimal configuration, with the lowest AICc value of 2739.13.

### 2.4 Model Evaluation and Analysis

Variable importance was evaluated using permutation importance percentages and jackknife test gain values, while model performance was assessed by computing the area under the receiver operating characteristic curve (AUC) (Phillips et al. 2017). The AUC value is a key metric for assessing model performance because it is not influenced by threshold selection. To further evaluate the predictive performance of the MaxEnt model beyond the conventional AUC, we incorporated the True Skill Statistic (TSS) and Cohen's Kappa coefficient as additional metrics (Allouche et al. 2006). A logistic threshold of 0.277, corresponding to the maximum test sensitivity plus specificity, was used to binarize the model outputs. Based on the confusion matrix, we calculated the TSS and Kappa values. The detailed data used for evaluation are provided in Table S4. Model performance was categorized as follows: failing (0.5–0.6), poor (0.61–0.7), fair (0.71–0.8), good (0.81–0.9), and excellent (0.91–1) (Phillips et al. 2006, 2017).

The jackknife test was employed to evaluate the significance and impact of different environmental variables on the species' spatial distribution (Phillips et al. 2004). A higher percentage contribution (PC) or permutation importance (PI) value of environmental factors indicates a greater impact on the model's predictions. The response curves of each variable can be specifically analyzed to determine the relationship between survival probability and specific environmental factors.

### 2.5 Suitability and Change Assessment

The probability threshold of “maximum sensitivity plus specificity” was selected to differentiate between suitable and unsuitable habitats for the species. This threshold is considered one of the most effective for yielding precise predictions and is commonly used in modeling research (Liu et al. 2005; Jiménez‐Valverde and Lobo 2007). To generate binary presence/absence maps and evaluate model performance using TSS and Kappa, we selected the threshold corresponding to the maximum test sensitivity plus specificity, which yielded a logistic value of 0.277. Habitat suitability was then classified into four levels: unsuitable (below 0.277), low suitability (0.277–0.5), medium suitability (0.5–0.7), and high suitability (0.7–1.0).

To analyze habitat changes between present and future scenarios, the “logistic” output from MaxEnt was transformed into binary maps using ArcGIS. Habitats above the threshold were classified as suitable, while those below were deemed unsuitable. Comparisons of current and future scenarios were made pairwise to create maps showing stable areas, expansions, and contractions. Potential distribution shifts, including areas of contraction, expansion, and stability regions, were assessed by SDMtoolbox 2.0 (Brown et al. 2017). The distribution was summarized as a single point, known as the niche centroid, which represents the overall distribution trend under different climate scenarios (VanDer Wal et al. 2013; Brown 2014). The centroid is calculated by averaging the coordinates of all “suitable” grid cells in the prediction model. Centroid analysis illustrated the shifts in the distribution of centroids under projected future climate scenarios. The centroid shift distance was calculated as the straight‐line distance between two points, using the Euclidean distance formula, based on the coordinates of the two centroids (Brown et al. 2017).

## 3 Results

### 3.1 Predicted Potential Distribution

**FIGURE 2:**
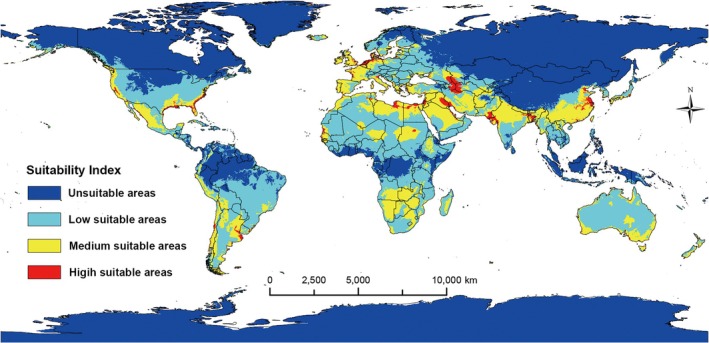
Predicted potential distribution of *Trogoderma granarium* under current climate. The blue areas represent unsuitable (with present probability of 0–0.277), the green areas represent low suitability (0.277–0.5), the yellow areas represent medium suitability (0.5–0.7), and the red areas represent high suitability (0.7–1.0).

Under current climatic conditions, the predicted potential geographic distribution of *T. granarium* is shown in Figure 2. The Khapra beetle ( *Trogoderma granarium* ) has suitable habitats across six continents. In North America, it is mainly distributed in the USA and Mexico, with medium and high suitability areas located along the U.S. coastal regions and western Mexico. In South America, the pest inhabits nearly the entire continent except for the Amazon rainforest, with medium and high suitability areas mainly in eastern Argentina and Chile. In Africa, suitable habitats span most of the continent, excluding some equatorial regions, with medium and high suitability concentrated in Mediterranean coastal countries like Egypt and Libya. In Europe, nearly all areas are suitable, with medium and high suitability found in Mediterranean coastal regions and countries such as Germany, Denmark, the Netherlands, Belgium, France, Spain, Portugal, Ireland, and the United Kingdom. In Asia, many medium and high suitability areas are located between 25° N and 35° N, particularly in southern China, northern India, Iran, Saudi Arabia, Iraq, Jordan, Syria, and regions near the Caspian Sea. In Oceania, *T. granarium* is distributed throughout Australia and New Zealand, primarily exhibiting medium to high suitability in coastal regions.

**FIGURE 3:**
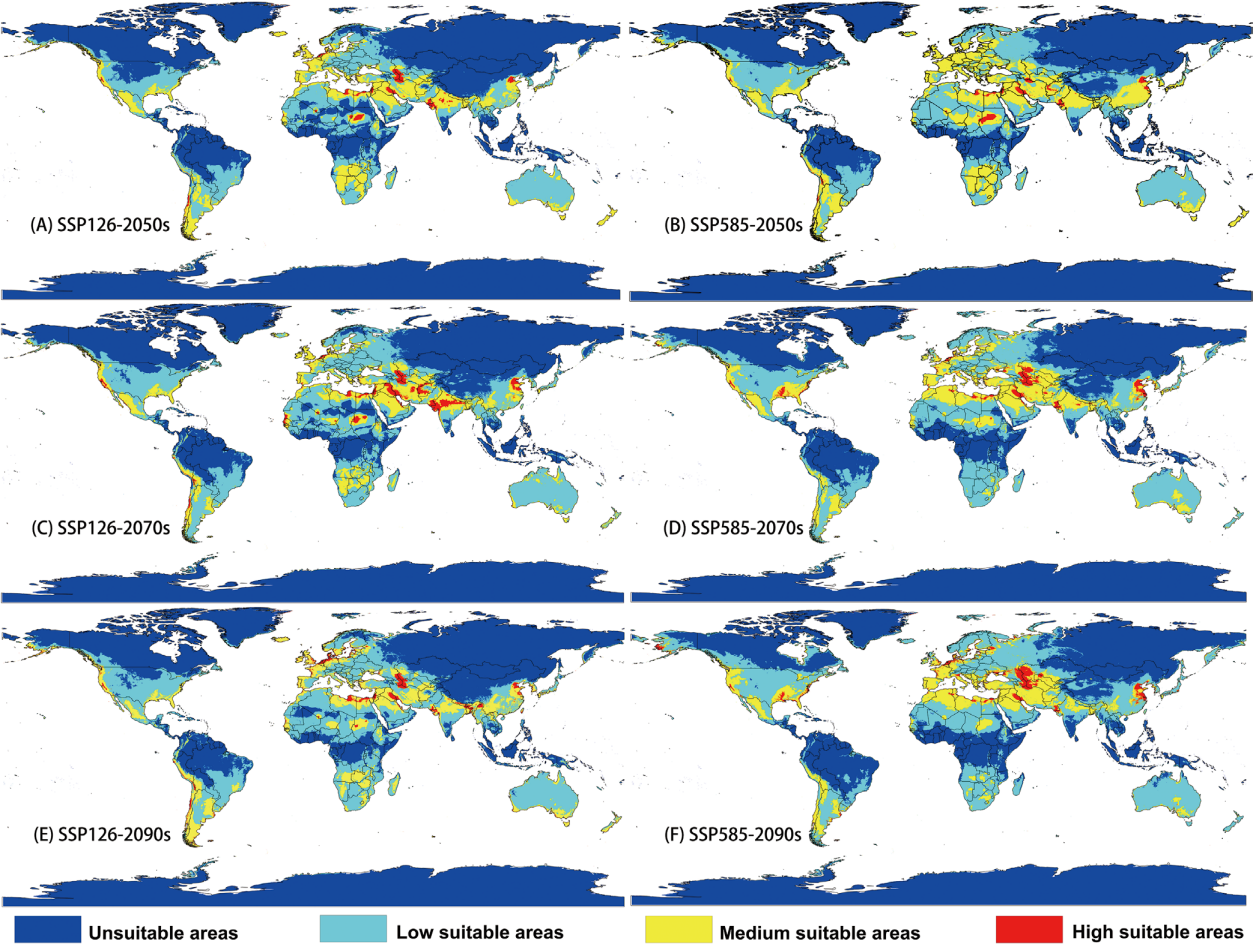
Predicted potential distribution of *Trogoderma granarium* under future climate: (A) in 2050s under scenario SSP126, (B) in 2050s under scenario SSP585, (C) in 2070s under scenario SSP126, (D) in 2070s under scenario SSP585, (E) in 2090s under scenario SSP126, (F) in 2090s under scenario SSP585.

Figure 3 shows the suitable habitat areas for this pest in the 2050s, 2070s, and 2090s under the SSP126 and SSP585 scenarios. Changes in medium and high suitability areas are observed in the current distribution, but the total suitable area remains relatively constant across different periods. In contrast, the SSP585 scenario for 2090 predicts a notable reduction in suitable habitat. In the 2050s, highly suitable areas decrease under both SSP126 and SSP585 scenarios, with a greater reduction under the latter. In the 2070s, new highly suitable areas are projected to emerge in the Middle East and India. By the 2090s, under the SSP585 scenario, highly suitable habitats are expected to become more concentrated in Central Asia.

### 3.2 Change in Potential Distribution Under Future Environmental Scenarios

**FIGURE 4:**
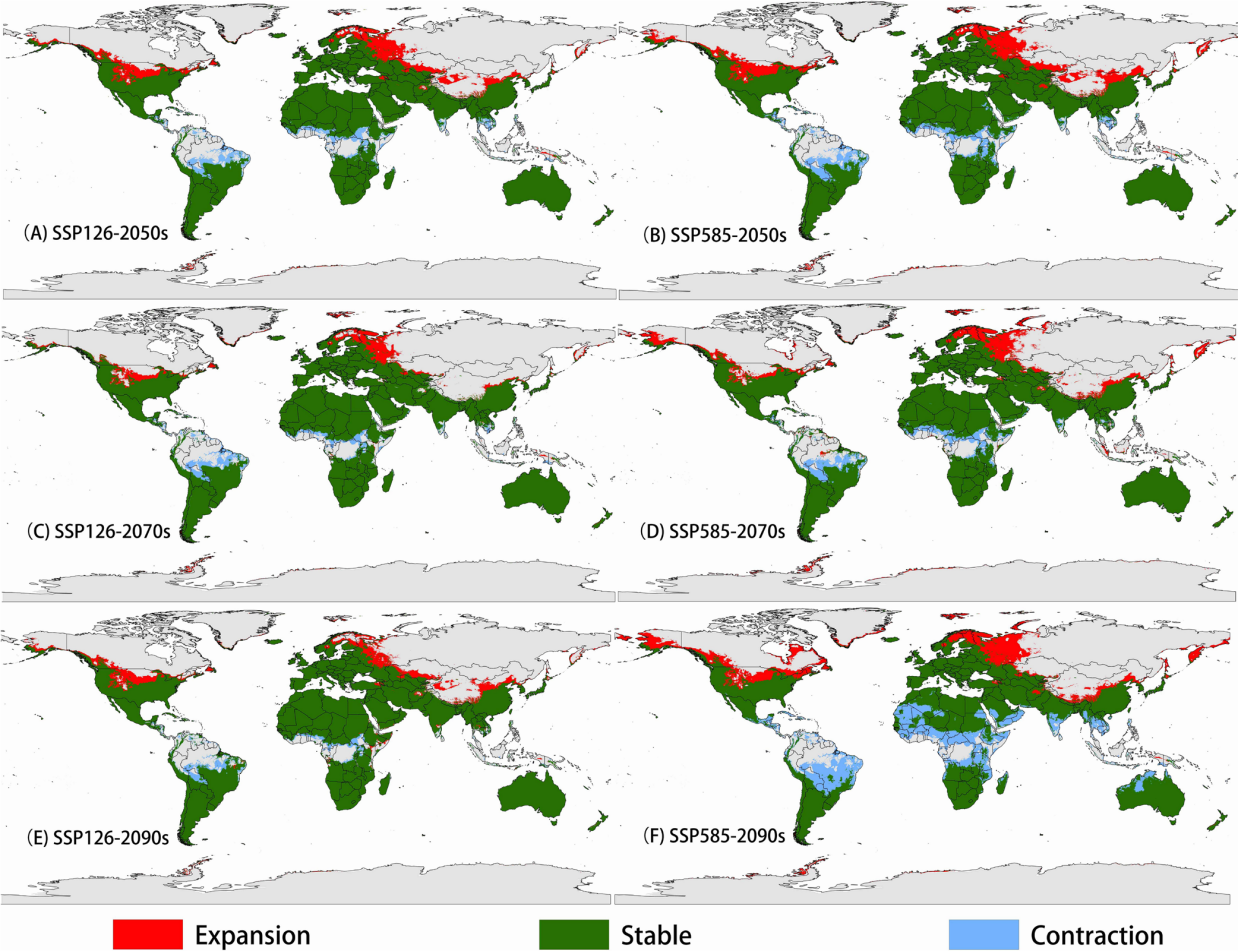
Predicted changes in modeled range of *Trogoderma granarium* under future climate. Binary models were generated based on “Maximum sensitivity plus specificity” threshold (0.277). Red color represents current unsuitable areas will become suitable areas in the future (i.e., expansion), green color represents the suitable areas will not change in the future, and blue color represents the current suitable areas of *T. granarium* will not be (i.e., contraction).

We analyzed potential shifts in the distribution range of *T. granarium* resulting from climate change by comparing maps of its current distribution with those projected for future scenarios (Figure 4). The results indicate that the species' potential range could expand or contract significantly in specific regions. Under various climate change scenarios, primary expansion areas are projected to include the northern United States, western Russia, southern Kazakhstan, and northern China. Regions predicted to remain stable show minimal changes from the current distribution. In contrast, primary contraction areas are expected in equatorial countries, including parts of Brazil and Central African nations. Notably, under the SSP585 scenario for the 2070s, contraction areas are projected to increase significantly compared to other scenarios.

The suitable habitat area shows a slight increase across different periods under the SSP126 scenario (Table 2). The potential distribution area of *T. granarium* is projected to expand by 0.95%, reaching 81.69 million km^2^, with an expansion rate of 9.10% and a contraction rate of 8.15% by the 2050s. The potential distribution area is expected to decrease by 1.26% to 79.90 million km^2^, with an expansion rate of 5.10% and a contraction rate of 6.36% by the 2070s. The suitable habitat area is projected to increase to 83.90 million km^2^, with an expansion rate of 8.05% and a contraction rate of 4.36% by the 2090s.

**TABLE 2:** Changes in the distribution of *Trogoderma granarium* are based on binary distribution projections in current and future scenarios.

Period	Climate scenario	Habitat area (×10^6^ km^2^)	Expansion (×10^6^ km^2^)	Unchanged (×10^6^ km^2^)	Contraction (×10^6^ km^2^)	Range change (%)	Expansion percentage (%)	Contraction percentage (%)
Current		80.91						
2050s	SSP126	81.69	7.36	7.43	6.59	0.95	9.10	8.15
	SSP585	81.60	9.22	7.24	8.53	0.86	11.40	10.54
2070s	SSP126	79.90	4.13	7.58	5.14	−1.26	5.10	6.36
	SSP585	81.93	7.20	7.47	6.18	1.26	8.90	7.64
2090s	SSP126	83.90	6.52	7.74	3.53	3.70	8.05	4.36
	SSP585	70.61	9.98	6.06	2.03	−12.73	12.34	25.06

The potential distribution area of *T. granarium* is predicted to slightly expand in the 2050s and 2070s but experience an apparent contraction in the 2090s under the SSP585 scenario (Table 2). In the first two periods, the expansion rates are 11.40% and 8.90%, respectively, with contraction rates of 10.54% and 7.64%. This will increase the potential distribution range by 0.86% and 1.26% in the 2050s and 2070s, respectively. In the 2090s, the expansion rate is projected to be 12.34%, with a contraction rate of 25.06%, leading to an overall decrease in the total distribution range by 12.73%, with a total suitable habitat area of 70.61 million km^2^.

### 3.3 Centroid Shifts

**FIGURE 5:**
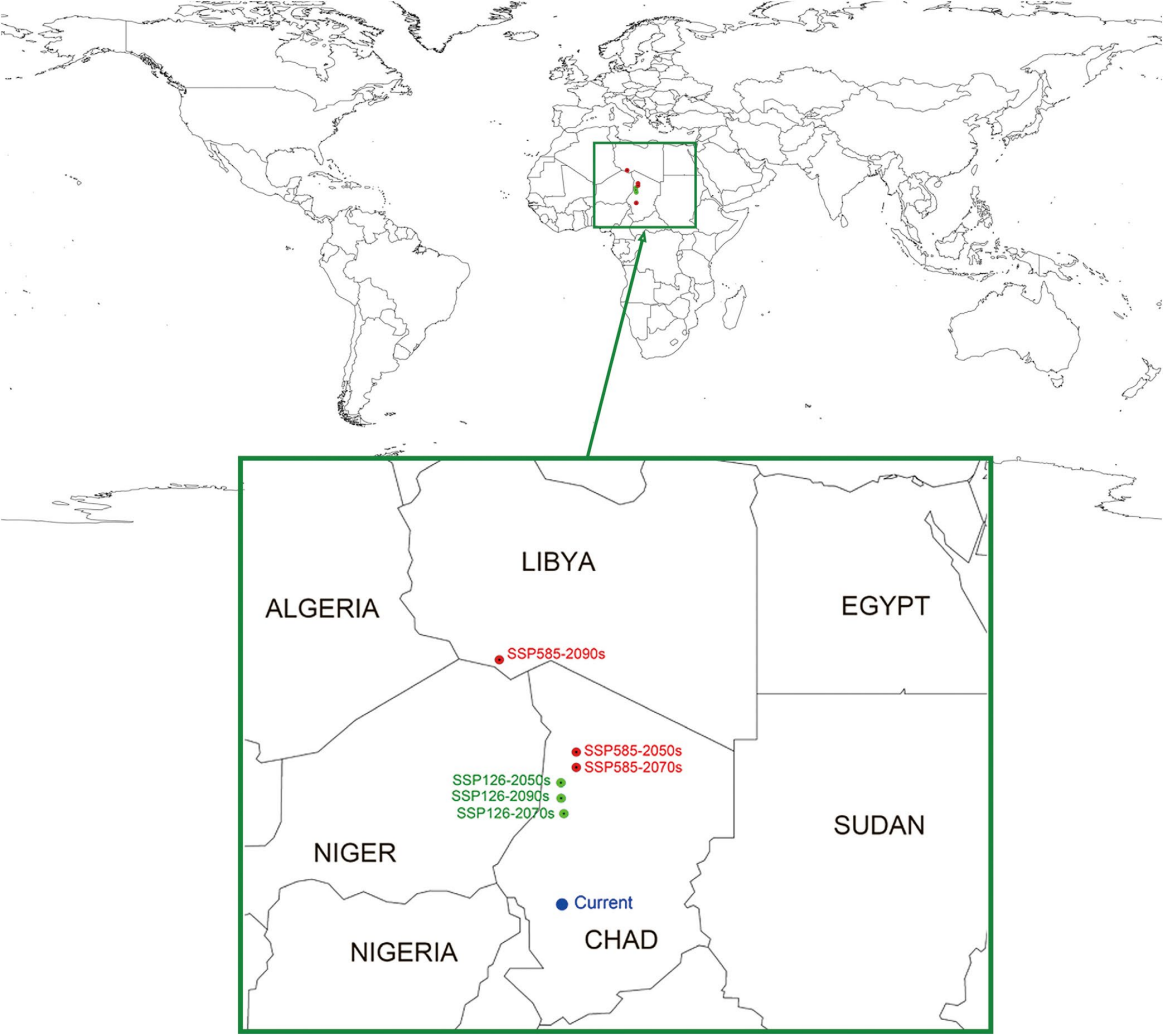
Centroid change in the potential distribution of *Trogoderma granarium* during 2050s, 2070s, and 2090s. The centroid is calculated by averaging the coordinates of all “suitable” grid cells in the prediction model. The blue color represents the centroid position of this species' suitable distribution under current climate conditions. The green color indicates the centroid positions at different periods under the SSP126 scenario, while the red color shows the centroid positions at different periods under the SSP585 scenario. Under future climate conditions, the centroid positions shift northward to varying extents compared to the current conditions, indicating a gradual northward expansion of this species.

We analyzed the potential geographic shifts of *T. granarium* at different time points by calculating the centroid of the distribution area (Figure 5). The results show that under the present climate conditions, the centroid of *T. granarium* is situated near 13° N, 26° E, in Chad. However, in future decades and climate scenarios, the centroid shifts northward to varying degrees. Under the SSP126 scenario, the centroid shifts northward by approximately 550–650 km. In contrast, under the SSP585 scenario, the northward shift is more pronounced, with the farthest shift occurring in the 2090s, reaching about 1250 km to approximately 23° N, 23° E. This centroid shift suggests that with climate warming, the suitable habitat may expand to higher latitudes, increasing the invasion risk for some Northern Hemisphere countries or regions.

### 3.4 Variable Importance

**FIGURE 6:**
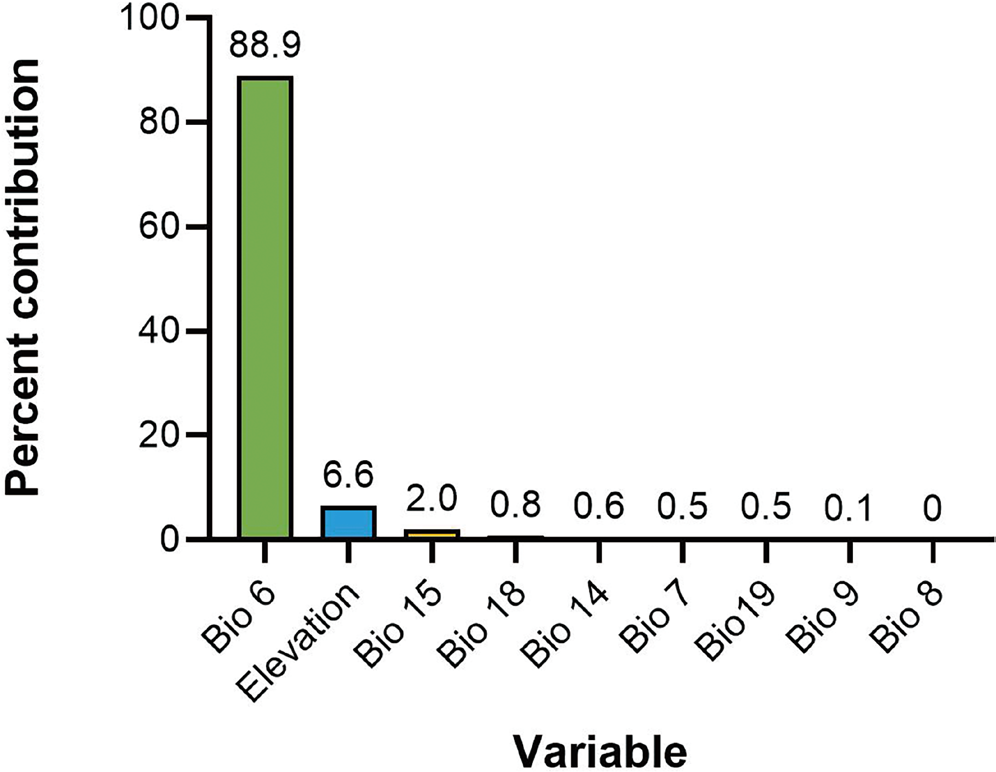
Percent contribution of environmental variable used in the modelling. A higher percentage indicates a greater contribution and influence on the model predictions. Among them, bio6, elevation, and bio15 are the three most influential factors on the model.

**FIGURE 7:**
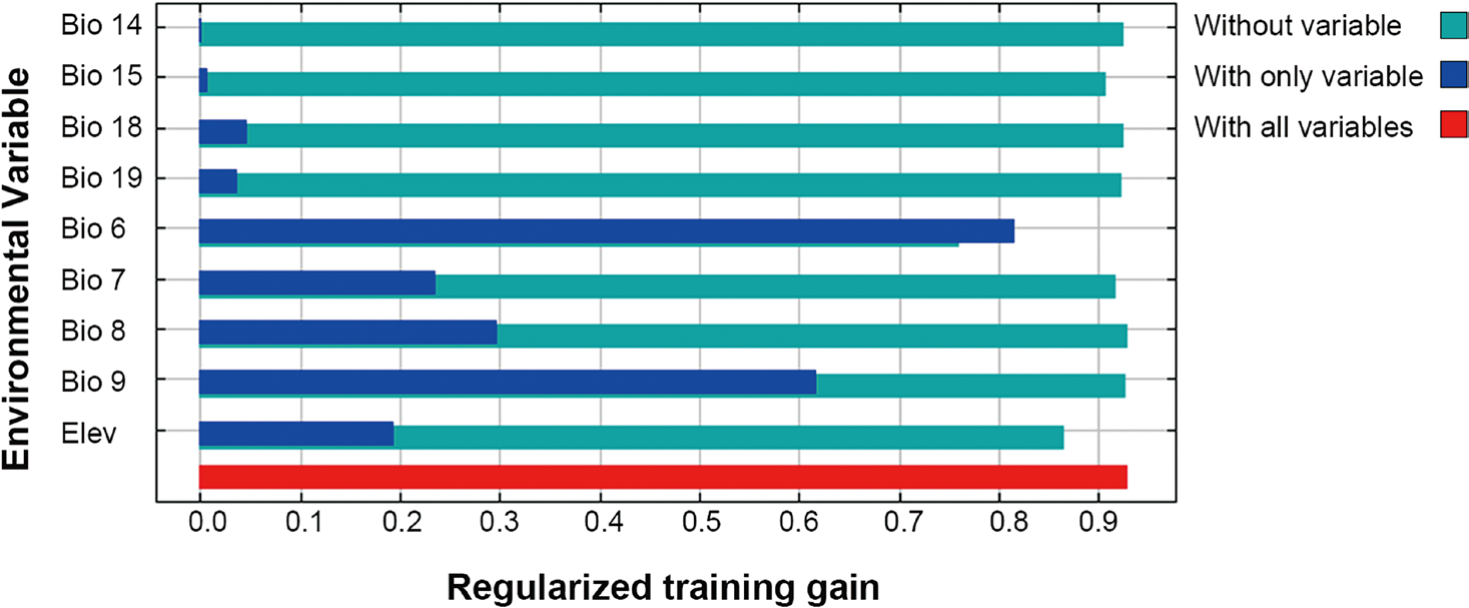
Results of jack‐knife tests. This figure displays the results of jackknife tests assessing the importance of each environmental variable in the MaxEnt model. The bars represent different variables' contributions, indicating their individual predictive power and relevance when used alone and in combination with other variables. Higher values suggest a greater influence of a specific variable on the model's performance, highlighting key factors that affect the species' distribution predictions.

The percentage contribution (Figure 6) and jackknife results (Figure 7) were used to assess the relative importance of the nine variables in the modeling. Among the variables, Bio6 (minimum temperature of the coldest month) has a significant impact on the prediction modeling, with a contribution rate of 88.9%, followed by elevation and Bio15 (precipitation seasonality) with contribution rates of 6.6% and 2.0%, respectively (Figure 6). In contrast, Bio18 (precipitation of the warmest quarter, 0.8%), Bio14 (precipitation of the driest month, 0.6%), Bio7 (annual temperature range, 0.5%), Bio19 (precipitation of the coldest quarter, 0.5%), and Bio9 (mean temperature of the driest quarter, 0.1%) each contribute less than 1%. Although the contribution of Bio8 was 0, it was retained because its value was not zero in the prediction test.

The variable importance pattern revealed by the jackknife test aligns closely with the percentage contribution results, particularly in identifying the most important variables (Figure 7). The jackknife test results showed that Bio6 provided the most valuable information when used independently. When Bio6 is omitted, the gain decreases the most, suggesting it contains the significant information that is not present in other variables. Bio7, Bio8, and Bio9 rank second to fourth, which is inconsistent with the percentage contribution analysis. Furthermore, when Bio7, Bio8, and Bio9 are excluded, the total contribution of the remaining variables is nearly equal to that of the complete set, suggesting that their information may be partially reflected by other factors in the model.

### 3.5 Response to Environmental Variables

**FIGURE 8:**
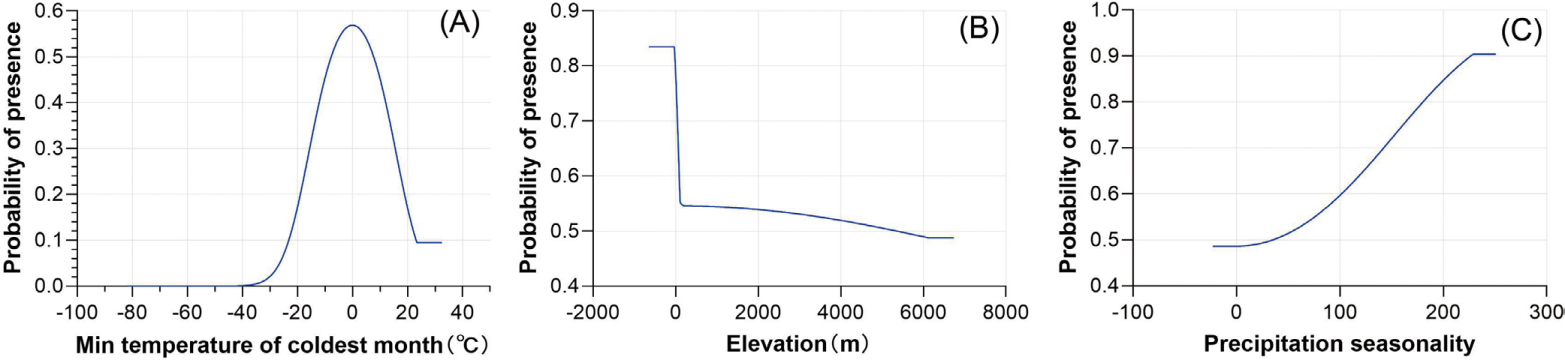
Response curves of the top environmental variables that contributed to the modelling. (A) Minimum temperature of the coldest month. (B) Elevation. (C) Precipitation seasonality. Each curve illustrates the predicted suitability for the species across the range of values for each variable, and higher suitability values indicate conditions more favorable for the species' presence.

Figure 8 shows the probability of *T. granarium* occurrence in relation to changes in the top three contributing environmental variables. The response curve for the minimum temperature of the coldest month (Bio6) demonstrates that presence probability increases from nearly 0 when Bio6 is around −30°C, peaks at approximately 0.57 near 0°C, and then declines, stabilizing above 23°C. However, this does not imply that the species can survive in subfreezing climates. Rather, it likely reflects tolerance to transient cold events in semi‐arid regions, particularly in human‐regulated storage environments where temperature extremes are buffered. Bio6 only represents a single month's minimum and does not capture the full thermal regime, so areas with low Bio6 values may still offer suitable conditions for part of the year. For elevation, the predicted presence probability is highest (~0.8) at slightly negative elevations, sharply declines to 0.48 at 100 m, and gradually decreases at higher elevations. This elevated suitability at negative elevations likely arises from occurrence records near coastal port cities and may partly reflect spatial biases or resolution limits in the elevation dataset, rather than true biological preference. For Bio15 (precipitation seasonality), presence probability increases from 0.49 to a peak of 0.91 at a value of 230. This is consistent with the species' ability to survive under variable moisture conditions, though extreme seasonality values should be interpreted cautiously due to potential data limitations. Overall, the pest shows a modeled preference for environments with moderate cold‐month temperatures (−10°C to 10°C), significant precipitation variability, and low altitudes—conditions commonly associated with warm, dry, human‐influenced storage environment.

## 4 Discussion

### 4.1 Predictive Performance and Model Robustness

In recent years, *T. granarium* has been frequently intercepted in many countries as a significant international quarantine pest, posing a substantial risk of spread. This study employed the MaxEnt model to forecast the potential geographic distribution of *T. granarium* . The AUC value of our predictive model is 0.89, indicating high predictive capability. Under the selected threshold, the model achieved a TSS value of 0.718 and a Kappa coefficient of 0.073 on the test dataset. The sensitivity and specificity were 0.869 and 0.849, respectively, indicating good discriminative performance in distinguishing between suitable and unsuitable habitats. The predicted suitable habitat range for *T. granarium* is extensive, covering almost all continents and encompassing nearly all surveyed records. Previous experience suggested that the species could be suitable for survival between latitudes 35° N and 35° S (Athanassiou et al. 2019). However, this study predicts that the species is potentially distributed across a wider latitudinal range, extending up to 60° N and down to 55° S. This finding exceeds the commonly reported range between 35° N and 35° S and may result from model extrapolation in regions where occurrence records are limited. Alternatively, it could signal potential future risks under extreme warming scenarios, where higher‐latitude environments may gradually become climatically suitable for *T. granarium* . The areas with medium to high suitability are primarily concentrated in the Northern Hemisphere, encompassing numerous countries, including the United States, China, Australia, South American nations, and most of Europe. The predicted information offers important guidance for countries and relevant authorities to develop monitoring and control strategies for *T. granarium* .

Compared with multispecies studies such as Cao and Feng (2024), our work offers greater methodological refinement, including model tuning, AICc‐based selection, and multi‐metric evaluation (TSS, Kappa, AUC). Importantly, we extend beyond broad trends by conducting region‐specific assessments and quantifying distributional risks in key countries, particularly in the Middle East, South Asia, and Africa. Our targeted recommendations tailored to agro‐ecological contexts offer more actionable insights than global overviews, thereby enhancing the ecological realism and practical relevance of species distribution modeling.

### 4.2 Environmental and Ecological Drivers of Distribution

In previous studies predicting the effects of global warming on species distribution, different results have shown that the range of species has expanded, shifted, or contracted (Tepa‐Yotto et al. 2021; Jin et al. 2022; Zhang et al. 2022; Lin et al. 2024). Under the future scenarios, the suitable area of *T. granarium* has shown changes, ranging from an increase of 3.70% to a decrease of −12.73%. The relationships between emissions and concentrations exhibit inconsistencies because Shared Socioeconomic Pathways are derived from different integrated assessment models. These discrepancies can make it challenging to interpret the resulting climate impacts (Meinshausen et al. 2020). These factors may collectively lead to variations in distribution growth rates of *T. granarium* under different climate scenarios. Wang et al. (2023) found that suitable areas for western flower thrips did not show consistent trends over time and carbon emissions; in the high emission scenario of the 2050s, there was an increase in suitable areas, while other climate scenarios showed a decrease. Except for the SSP585 scenario, where the total suitable habitat area of *T. granarium* did not change significantly, there was some regional shift. In various periods, the suitable habitat area near the equator decreased, while the expansion primarily occurred in the northern regions of the previously suitable areas in the Northern Hemisphere. Zhao et al. (2023) found that the geographic range of the large black grain beetle would significantly expand northward, with the RCP8.5 (Representative Concentration Pathway 8.5) showing the largest expansion, suitable areas increasing in the Northern Hemisphere. The results of this study using similar methods show similarities with ours. The different SSP scenarios may also indirectly influence the future distribution of *T. granarium* through ecological and anthropogenic pathways. For instance, SSP585 represents a fossil‐fuel‐driven, resource‐intensive trajectory, potentially increasing global grain production and storage density, thereby expanding host availability. It may also promote greater transboundary trade and movement of stored products, enhancing opportunities for pest dispersal. In contrast, SSP126 reflects a more sustainable development path with stricter biosecurity policies, reduced emissions, and improved storage practices, which could constrain pest expansion. These interacting socio‐environmental dynamics should be considered when interpreting distribution shifts under different climate scenarios.

The MaxEnt model identified two bioclimatic variables and elevation as the primary factors influencing the potential distribution of *T. granarium* . Notably, the minimum temperature of the coldest month emerged as a crucial variable impacting its distribution. The high contribution rate of Bio6 indicates that the species is highly sensitive to cold climates. The minimum temperature of the coldest month may limit the survival and reproduction of *T. granarium* . Extreme low temperatures may reduce egg hatching rates or increase larval mortality. Xie et al. (2023) also identified Bio6 as the most critical environmental factor in their study using MaxEnt for species distribution. In addition to climate factors, the contribution of elevation is also significant, indicating that the distribution of *T. granarium* is closely related to topography. Elevation not only affects temperature but also influences other climatic conditions such as precipitation and humidity, which collectively determine the suitable habitat for *T. granarium* . This study shows that the species is suitable for low‐altitude areas. Other insect taxa may also show high correlation with elevation. MaxEnt highlights the influence of temperature fluctuations and weather events typically associated with high‐altitude environments on the distribution of 10 Italian endemic insects (Urbani et al. 2017). Additionally, the high contribution rate of Bio15 is relatively high but does not indicate a high sensitivity of *T. granarium* to precipitation fluctuations. The species is known to infest harvested food with moisture content as low as 2%, thriving across a range of temperatures. It can remain concealed within storage facilities, sometimes overwintering for several months or even years (Ahmedani, Khaliq, et al. 2007; Mason and McDonough 2012). Severe fluctuations in precipitation may lead to unstable food sources affecting the population dynamics of *T. granarium* , but the species can maintain its population through drought tolerance. In addition to climatic factors, the establishment of *T. granarium* is closely tied to its ecological traits. The species thrives in hot, dry environments and can reproduce under extremely low humidity. Its larvae can enter long‐term diapause and survive prolonged starvation, enhancing its persistence in storage conditions. However, high humidity may suppress its populations by favoring competing species, thus influencing actual establishment despite climatic suitability (Banks 1977).

### 4.3 Future Risk and Management Implications

Although *T. granarium* is widely recognized as a significant stored‐product pest, its ability to establish in truly natural environments is limited. The species depends heavily on human‐associated storage systems that provide stable high temperatures, low humidity, and a continuous food supply. In the wild, it rarely completes its life cycle due to fluctuating conditions and lack of food (Banks 1977). Occasional survival has been observed in transitional habitats near ports or damaged storage sites, but these are extensions of artificial environments rather than true natural habitats. Therefore, its distribution remains tightly linked to human‐mediated dispersal and storage ecology. This study's results show some countries and regions that warrant attention and ongoing monitoring include China, Japan, Thailand, Laos, and Vietnam, Argentina, Chile, USA, Mexico, Australia, New Zealand, and most European countries. Some of these countries have previously reported infestations but are considered eradicated (Day and White 2016; Adler et al. 2022), while others have frequent interception reports (Hagstrum and Subramanyam 2009; Athanassiou et al. 2016). In these areas, if infections are not effectively controlled, they could lead to significant economic losses for the country's exports. Despite the potential risks, the EU has not classified this species as a quarantine or regulated pest under the latest regulations (EU) No 2016/2031. Adler et al. (2022) observed that EU authorities currently do not consider this storage pest a significant threat, at least not enough to warrant regulatory action. However, if the species spreads further north, it could pose serious risks to European agriculture and food trade. This study supports this view, as almost all of Europe is a suitable habitat for *T. granarium* under current and future scenarios. Under future conditions, although the suitable habitat range does not significantly expand, it shifts northward, with new suitable areas mainly in northern parts of the United States, western Russia, southern Kazakhstan, and northern China. This northward centroid shift implies elevated invasion risk in temperate zones, where climatic constraints are diminishing. Given that many of these regions have large grain reserves and trade networks, early warning and quarantine strategies should be prioritized to prevent establishment.

### 4.4 Model Limitations and Future Improvements

Although the 7 km spatial thinning threshold was primarily selected to reduce sampling bias and spatial autocorrelation, it also aligns with the ecological traits of *T. granarium* , a stored‐product pest with limited natural dispersal largely facilitated by human‐mediated transport. Despite the robustness of the optimized MaxEnt model, several sources of uncertainty should be acknowledged. The binary threshold (0.277) was chosen using the maximum sensitivity plus specificity criterion, but no extensive sensitivity analysis was conducted, potentially affecting the delineation of suitable habitats. Moreover, although two GCMs under distinct SSP scenarios were employed, incorporating additional climate models and ensemble approaches would enhance prediction stability and ecological inference. Importantly, while MaxEnt offers valuable insights into climatic suitability, its correlative nature means predictions must be interpreted with caution. As a strictly synanthropic species, *T. granarium* depends heavily on international trade, storage infrastructure, and quarantine enforcement. Thus, its actual distribution results from both bioclimatic suitability and anthropogenic drivers. For example, some model‐predicted suitable areas in Europe have historically eliminated infestations due to stringent regulation and advanced storage systems. Additionally, the use of presence‐only data may introduce bias, particularly when occurrence records are clustered around ports or trade centers. Despite spatial filtering, such bias can affect model transferability. Therefore, future pest risk assessments should integrate socio‐economic data—such as trade routes and biosecurity capacity—alongside climatic projections to improve reliability and policy relevance.

## 5 Conclusions

In recent years, *Trogoderma granarium* has been frequently intercepted in many countries as an internationally significant quarantine pest, posing a huge risk of spread. This study used the MaxEnt model to predict the current and future potential distribution of *T. granarium* and analyzed the future trends and key environmental factors influencing its distribution. The results show that countries currently without *T. granarium* distribution records, such as the United States and China, have extensive high‐suitability areas for this pest. European countries are generally suitable for the pest's survival, indicating a high risk of invasion, and this risk will continue to exist in the future with the suitable habitat shifting northward. The predicted distribution patterns and key regulatory factors provide important references for relevant countries or departments to develop monitoring and control policies for *T. granarium* .

## Author Contributions

**Chao Zhao:** conceptualization (equal), data curation (equal), funding acquisition (equal), investigation (equal), software (equal), writing – original draft (equal), writing – review and editing (equal). **Duangsamorn Suthisut:** funding acquisition (equal), investigation (equal). **Chunqi Bai:** data curation (equal), project administration (equal), software (equal), writing – review and editing (equal). **Lei Yan:** data curation (equal), project administration (equal), software (equal), writing – original draft (equal). **Dianxuan Wang:** funding acquisition (equal), investigation (equal). **Liang Chen:** funding acquisition (equal), investigation (equal). **Jianhua Lü:** funding acquisition (equal), investigation (equal). **Liang Li:** funding acquisition (equal), investigation (equal). **Peng Li:** conceptualization (equal), funding acquisition (equal), investigation (equal), resources (equal), visualization (equal), writing – original draft (equal), writing – review and editing (equal).

## Conflicts of Interest

The authors declare no conflicts of interest.

## Supporting information


**Table S1:** ece372159‐sup‐0001‐TableS1.csv.

**Table S2:** ece372159‐sup‐0002‐TableS2.csv.

**Table S3:** ece372159‐sup‐0003‐TableS3.csv.

**Table S4:** ece372159‐sup‐0004‐TableS4.csv.

## Data Availability

I confirm that the Data Availability Statement is included in the main file of my submission, and that access to all necessary data files is provided to editors and reviewers. All the required data are uploaded as Supporting Information.
